# Application of Machine Learning Techniques for Clinical Predictive Modeling: A Cross-Sectional Study on Nonalcoholic Fatty Liver Disease in China

**DOI:** 10.1155/2018/4304376

**Published:** 2018-10-03

**Authors:** Han Ma, Cheng-fu Xu, Zhe Shen, Chao-hui Yu, You-ming Li

**Affiliations:** Department of Gastroenterology, The First Affiliated Hospital, College of Medicine, Zhejiang University, Hangzhou 310003, Zhejiang Province, China

## Abstract

**Background:**

Nonalcoholic fatty liver disease (NAFLD) is one of the most common chronic liver diseases. Machine learning techniques were introduced to evaluate the optimal predictive clinical model of NAFLD.

**Methods:**

A cross-sectional study was performed with subjects who attended a health examination at the First Affiliated Hospital, Zhejiang University. Questionnaires, laboratory tests, physical examinations, and liver ultrasonography were employed. Machine learning techniques were then implemented using the open source software Weka. The tasks included feature selection and classification. Feature selection techniques built a screening model by removing the redundant features. Classification was used to build a prediction model, which was evaluated by the F-measure. 11 state-of-the-art machine learning techniques were investigated.

**Results:**

Among the 10,508 enrolled subjects, 2,522 (24%) met the diagnostic criteria of NAFLD. By leveraging a set of statistical testing techniques, BMI, triglycerides, gamma-glutamyl transpeptidase (*γ*GT), the serum alanine aminotransferase (ALT), and uric acid were the top 5 features contributing to NAFLD. A 10-fold cross-validation was used in the classification. According to the results, the Bayesian network model demonstrated the best performance from among the 11 different techniques. It achieved accuracy, specificity, sensitivity, and F-measure scores of up to 83%, 0.878, 0.675, and 0.655, respectively. Compared with logistic regression, the Bayesian network model improves the F-measure score by 9.17%.

**Conclusion:**

Novel machine learning techniques may have screening and predictive value for NAFLD.

## 1. Introduction

Nonalcoholic fatty liver disease (NAFLD) is one of the most common chronic liver diseases worldwide and has become a significant public health concern [1, 2]. The spectrum of NAFLD ranges from simple steatosis and nonalcoholic steatohepatitis (NASH) to fibrosis. Simple steatosis is considered to have a benign progression, while NASH may progress to fibrosis, cirrhosis, and even hepatocellular carcinoma [3, 4]. Furthermore, NAFLD is a disease significantly associated with metabolic syndrome, cardiovascular disease, and type 2 diabetes [5–7]. For these reasons, it is critically important to obtain an early diagnosis that would enable improved prevention and management of NAFLD.

A liver biopsy is the gold standard for a NAFLD diagnosis. However, significant side effects and the susceptibility of this technique to sampling error raise the need for finding reliable diagnostic biomarkers of this disease [8, 9]. Ultrasonography is noninvasive, reasonably accurate, and widely used in the clinical diagnosis of NAFLD; however, it is not sensitive enough to detect mild steatosis [10]. Recent attention has been focused on finding surrogate markers of fatty liver [11–13]. The fatty liver index (FLI), which is a validated formula based on triglycerides, body mass index (BMI), gamma-glutamyltransferase (GGT), and waist circumference (WC), is widely used in many countries as an index of NAFLD biomarkers [11]. The ZJU index is a novel model for predicting NAFLD in the Chinese population [12]. The hepatic steatosis index (HSI) was also efficient for screening NAFLD, which is comprised of ALT, AST, BMI, gender, and history of diabetes [13]. However, when considering big data, the application of these current surrogate markers has not been well documented. In addition, these conventional statistical techniques are model-driven; they begin with a logistic regression model and then ascertain whether the data fit the suggested model [14, 15]. Validation is based on the accuracy of fit tests. This approach has proven itself over the years and is widely used in epidemiological research. However, it has limitations.

In the field of computer science, data mining, indicating the extraction of focused information from a larger data set, is a modern term describing this approach for analyzing big data sets [16, 17]. Machine learning (ML) algorithms are data-mining tools. Machine learning refers to a variety of techniques dealing with pattern recognition based on models for classification and the prediction of new data. In principle, ML has four steps: problem definition, data collection and preparation, model building, and model prediction. There are 11 state-of-the-art machine learning techniques [18–22], namely, logistic regression (LR), k-nearest neighbor (kNN), support vector machine (SVM), naïve Bayes (NB), Bayesian network (BN), decision tree (C4.5), AdaBoosting, bagging, random forest (RF), hidden naïve Bayes (HNB), and aggregating one-dependence estimators (AODE).

Here, we performed a cross-sectional study to investigate useful screening and predictive models for NAFLD by machine learning techniques.

## 2. Methods

### 2.1. Data Collection and Preparation

#### 2.1.1. Subjects

A cross-sectional study was performed with data from 10,508 participants who attended an annual health examination in the First Affiliated Hospital, College of Medicine, Zhejiang University, China, in 2010. Individuals with the following characteristics were excluded: alcohol consumption greater than 140 g/week for males and 70 g/week for females or a history of viral hepatitis, autoimmune hepatitis, or other form of chronic liver disease. Those who were previously diagnosed as either diabetic or anemic were also excluded. The definition of anemia in our study was serum hemoglobin <120 g/L for males and < 110 g/L for females. Verbal informed consent was obtained from each participant and was recorded by the physician who explained the study procedures. Within the informed consent, participants agree to publish the data collected from them. Written informed consent was not required due to the observational nature of the study, and we therefore verbally informed all participants about the study. Subject information was anonymized at the collection and analysis stage. The study protocol was approved by the Ethics Committee of the First Affiliated Hospital, College of Medicine, Zhejiang University, and was in compliance with the Helsinki Declaration. All methods were performed in accordance with the approved guidelines.

#### 2.1.2. Clinical Examination

The clinical examinations were performed as previously described [23]. In brief, all subjects were required to refrain from exercise for one day prior to the examination. Systolic and diastolic blood pressures were measured by standard clinical procedures. The standing height and body weight were recorded for all subjects. The body mass index (BMI) was calculated as the weight divided by height squared and was used as the criteria for the diagnosis of overweight and obesity.

Fasting blood samples were obtained for the analysis of biochemical variables and were not frozen. The variables included liver enzymes, lipids, uric acid, and glucose. All of the biochemical variables were measured by a Hitachi 7600 autoanalyzer (Hitachi, Tokyo, Japan) using standard methods.

#### 2.1.3. Diagnosis of NAFLD

The diagnosis of NAFLD was based on criteria from the Chinese Liver Disease Association [24]. An ultrasonic examination was carried out by a trained ultrasonographist who was unaware of the results of the physical examination and biochemical analyses. The examination was performed using a Toshiba Nemio 20 sonography machine with a 3.5 MHz probe (Toshiba, Tokyo, Japan).

### 2.2. Conventional Statistical Techniques

Statistical analyses were performed using SPSS 13.0 for Windows (SPSS, Chicago, IL). The Kolmogorov-Smirnov test was used to assess whether continuous data were normally distributed. Continuous variables are expressed as the mean and standard deviation (SD) or the median and interquartile range and were compared with the Student's t-test or the Mann–Whitney U test. The chi squared test was used for the comparison of categorical variables. The stepwise logistic regression analysis (Backward: Wald; Entry: 0.05, Removal: 0.10) was used to evaluate the risk factors for NAFLD. A value of P <0.05 (2-tailed test) was considered to be statistically significant.

### 2.3. Machine Learning Techniques

Machine learning techniques were implemented using the Weka open source software. Weka is a collection of machine learning algorithms for data-mining tasks that can be applied directly to a data set or be used in one's own Java code. Weka contains tools for data preprocessing, classification, regression, clustering, association rules, and visualization. It is also used for developing new machine learning schemes.

Based on data given as a set of attributes that are assigned to a specific predefined class, the machine learning technique tasks included feature selection techniques and classification techniques, referring to screening model and prediction model building, respectively. The feature selection techniques built a screening model by removing redundant features. Classification was used to build a prediction model, which was evaluated by the F-measure.

#### 2.3.1. Screening Model Building

Feature selection techniques were used to build a screening model. After removing the redundant features, discriminative features were then selected based on weight scores. Features are the various quantifiable characteristics of patients who can potentially differentiate patients who suffer from fatty liver disease from those that do not, and many features could be used in our study. In medicine, there are various methods to obtain these measures. In this study, we considered two types of features: basic features and advanced features. Basic features refer to those that can be collected from simple operations such as a clinical examination. Advanced features refer to those that can be collected through biochemical analysis (e.g., blood testing). By leveraging a set of statistical testing techniques, including 4 steps (correlation, redundancy analysis, “out-of-bag” estimation, and the Scott-Knot test), we extracted the top 5 features based on their information gain scores.

#### 2.3.2. Prediction Model Building and Evaluation

**Figure 1:**
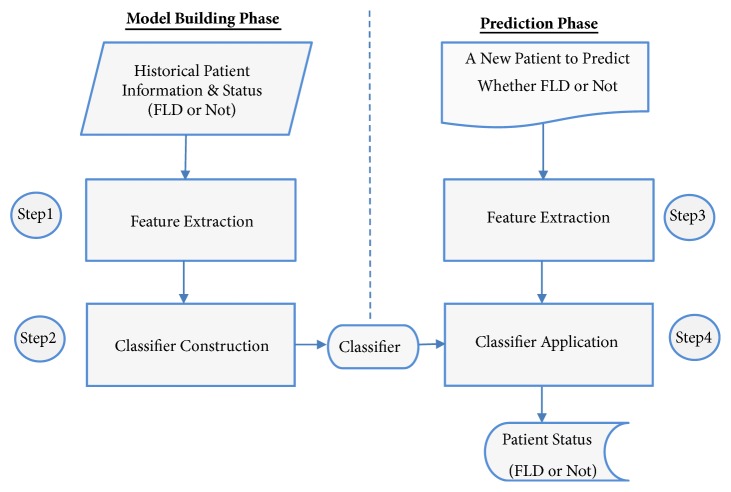
Overall framework for fatty liver disease (FLD) prediction.

Figure 1 shows our overall framework for fatty liver disease (FLD) prediction. The entire framework includes two phases: a model building phase and a prediction phase. In the model building phase, our goal was to build a classifier from the historical patient information from individuals who have a known medical status (i.e., FLD patient or not). In the prediction phase, the classifier is applied to predict if a new patient would develop FLD or not. Our framework first extracts features from the set of historical patients (**Step 1**), and then it constructs a classifier based on features of the historical patients (**Step 2**). A classifier is a machine learning model which assigns labels (in our case: FLD or not) to a data point (in our case: a patient) based on its features [16]. The classifier construction step compares and contrasts the features of patients who have FLD and those of patients that do not. Various thresholds or rules would then be learned, and these are stored in the constructed classifier. There are various classification algorithms that can be used for this step. The goal of our study was to investigate the effectiveness of various algorithms to predict whether a patient had FLD. The algorithms are grouped into three families: traditional algorithms, ensemble algorithms, and algorithm extensions. The traditional classification algorithms include k-nearest neighbor (kNN) [16], support vector machine (SVM) [16], logistic regression (LR) [25], naïve Bayes [26], Bayesian network (BN) [27], and decision tree [28]. Notice that we leveraged the K2 algorithm [29] for learning Bayesian network, which is the default algorithm used in Weka. The ensemble classification algorithms include adaptive boosting (AdaBoost) [30], bootstrap aggregating (bagging) [31], and random forest [32, 33]. The algorithm extensions include hidden naïve Bayes (HNB) and aggregating one-dependence estimators (AODE) [19–22].

In the prediction phase, the classifier that was constructed in the model building phase is then used to predict whether a patient with unknown label would have FLD. For each unknown patient, we first extracted the features (**Step 3**). The features extracted from the patients are the same as those used in the model building phase. We then input the features to the classifier in the classifier application step (**Step 4**). This step then gives the prediction result, which is either FLD or not FLD.

#### 2.3.3. Statistical Analyses

*Experiment Setup*. To simulate the practical usage of FLD prediction, we used a 10-fold cross-validation process to evaluate the 11 classification algorithms [16, 19, 20]. We implemented the 11 classification algorithms using Weka [34]. Each of the 11 algorithms used one or more parameters. For kNN, we set the number of neighbors to 5. For SVM, we set the kernel as a normalized polynomial kernel. For logistic regression, naïve Bayes, Bayesian network, C4.5, hidden naïve Bayes (HNB), and aggregating one-dependence estimators (AODE), we used the default Weka settings. For AdaBoost and bagging, we used C4.5 as the base classifier and iterated the entire process 10 times. For random forest (RF), we set the number of trees as 10 and used C4.5 as the base decision tree.

*Definition of Evaluation Index*. According to methods used in prior studies [16], we computed the accuracy, specificity, precision, recall (i.e., sensitivity), and the F-measure to evaluate the performance of the different FLD prediction algorithms. There are four possible outcomes for a patient: a patient (i.e., an instance) can be predicted to have FLD when he truly has FLD (true positive, TP); he is predicted to have FLD when he actually does not have FLD (false positive, FP); he is predicted not to have FLD when he truly has FLD (false negative, FN); or he is predicted not to have FLD when he actually does not have FLD (true negative, TN). Based on these possible outcomes, the accuracy, precision, recall, and F-measure were defined as follows:

*Accuracy.* The proportion of instances that are correctly labeled among the total number of instances [35].

(1) $$\begin{array}{c} P=\frac{\left(TP+TN\right)}{\left(TP+TN+FP+FN\right)} \end{array}$$

*Specificity*. The proportion of instances predicted not to have FLD and are correctly identified as such.

(2) $$\begin{array}{c} P=\frac{TN}{\left(TN+FP\right)} \end{array}$$

*Precision.* The proportion of instances that are correctly predicted to have FLD among those labeled FLD [36].

(3) $$\begin{array}{c} P=\frac{TP}{\left(TP+FP\right)} \end{array}$$

*Recall (Sensitivity).* The proportion of instances FLD that are correctly labeled [37].

(4) $$\begin{array}{c} R=\frac{TP}{\left(TP+FN\right)} \end{array}$$

*F-Measure.* A summary measure that combines both precision and recall. It evaluates whether an increase in precision (recall) outweighs a reduction in recall (precision) [34].

(5) $$\begin{array}{c} F=\frac{\left(2\times P\times R\right)}{\left(P+R\right)} \end{array}$$

Following prior studies [38–40], we took advantage of KEEL Data-Mining Software Tool [41] to perform Wilcoxon signed-rank tests to compare the F1-measures for each pair of algorithms.

## 3. Results

### 3.1. Characteristics of Study Participants and Results from a Conventional Statistical Technique

Among the 10508 enrolled subjects, 2522 (1907 males and 615 females) met the diagnostic criteria for NAFLD. The prevalence of NAFLD was 24.00% (27.73% and 16.94%, for males and females, respectively).

The characteristics of the participants, classified by the presence or absence of NAFLD, are presented in Table 1. Subjects with NAFLD are typically older, male, and had higher values for BMI, glutamic-pyruvic transaminase (ALT), glutamic oxaloacetic transaminase (AST), alkaline phosphatase (ALP), gamma-glutamyl transpeptidase (*γ*-GT), total bilirubin (TB), direct bilirubin (DB), total cholesterol, triglycerides, LDL cholesterol, fasting plasma glucose, serum uric acid, and a lower HDL cholesterol level than subjects without NAFLD.

Logistic regression analysis was applied to explore the risk factors for NAFLD. Variables including age, gender, weight, height, BMI, ALT, AST, ALP, *γ*-GT, total cholesterol, triglycerides, HDL and LDL cholesterol, fasting plasma glucose, and serum uric acid were entered into the analysis. Our results showed that 11 of the variables remained in the final equation (Table 2), suggesting that they were significantly associated with the risk for NAFLD. Specifically, those variables are independent risk factors for the presence of NAFLD, and ALT, triglycerides, age, HDL, and glucose are the five top factors affecting NAFLD, according to the score Wald *χ*^2^ in Table 2.

However, the results from the logistic regression analysis had limitations. It was unclear as to which approach had a better calibration and minimized the errors between the predicted values and the real data. Thus, machine learning techniques were introduced.

### 3.2. Screening Model

In total, we had 4 basic features (age, gender, height, and weight). The advanced features refer to those that could be collected via biochemical analysis (i.e., blood testing). We collected 15 features from the blood test. The BMI was calculated using the height and weight. In total, we collected 20 different features to help users to identify whether a patient had FLD. We also extracted the most discriminative features from the larger set of 20 features. By leveraging a set of statistical testing techniques, including 4 steps (correlation, redundancy analysis, “out-of-bag” estimation, and the Scott-Knot test), the top 5 features contributing the most to NAFLD were found to be BMI, triglycerides, *γ*-GT, ALT, and uric acid. These features all had a medium or large positive effect on NAFLD at a 99% confident level.  Table 3 presents the top 5 discriminative features based on the information gain scores. It was clear that among the 20 features, the BMI, TG, ALT, GGT, and uric acid values were the most discriminative features.

### 3.3. Seeking the Best Prediction Model from among the 11 Algorithms

A 10-fold cross-validation process was used in the classification phase to evaluate the machine learning techniques. Subjects were randomly divided into 10 groups, 9 of which were used to build a prediction model, and the remaining group was used to evaluate the model. The entire process was repeated 10 times, and the average performance was recorded.

When employing 11 different classification algorithms to predict whether a patient had FLD, it is unsurprising that different algorithms would have different performances. Identifying which algorithms showed the best performance would help users to select quality FLD prediction algorithms. To clarify this, we ran each algorithm on the collected data set and recorded its accuracy, precision, recall, and F-measure scores.  Table 4 presents these scores for the 11 algorithms.

We found that for the different algorithms, the various classification algorithms showed different performances. Among the 11 algorithms, logistic regression (LR) achieves the best accuracy values (83.41%), SVM achieves the best precision values (0.725), aggregating one-dependence estimators (AODE) achieves the best recall values (0.680), and Bayesian network (BN) achieves the best F-measure values (0.655). The F-measure is the most important evaluation metric, which will be further explained in the discussion section. The results showed that of the 11 state-of-the-art machine learning techniques, the Bayesian network technique demonstrated the best overall performance. It achieved accuracy, specificity, sensitivity, and F-measure scores of up to 83%, 0.878, 0.675, and 0.655, respectively. Compared with logistic regression, Bayesian network showed a 9.17% improvement in the F-measure score.

We also calculated the accuracy, precision, recall, and F-measure scores for FLI and HIS, in order to compare their results with the machine learning algorithms, which are presented in Table 4. A FLI < 30 rules out hepatic steatosis and a FLI ≥ 60 confirms fatty liver [11]. A HSI of <30.0 rules out NAFLD, while a HSI of >36.0 confirms fatty liver [13]. As for FLI, We noticed that this method achieves a precision of 0.749, which is higher than all of the machine learning models. However, it achieves a much lower recall than the 11 machine learning models. We also noticed that FLI and HSI achieve an F-measure of 0.318 and 0.524, respectively. All of the machine learning models achieve higher F-measure than FLI and HSI. In particular, the Bayesian network model outperforms FLI and HSI by 105.97% and 25.00% in terms of F-measure, respectively.

Furthermore, we took advantage of KEEL Data-Mining Software Tool to perform Wilcoxon signed-rank tests to compare the F1-measures for each pair of algorithms. As a result, we noticed that Bayesian network (BN) algorithm statistically significantly outperforms the other algorithms in terms of F-measure with p-values < 0.05.

## 4. Discussion

In our study, 11 state-of-the-art machine learning techniques were investigated to evaluate the best clinical predictive model of NAFLD. The results from the screening model revealed the top 5 most discriminative features, based on information gain scores, to be weight, TG, ALT, GGT, and serum uric acid levels. Thus, in practice, users could focus on these 5 features. The results from the prediction model demonstrated that the Bayesian network model had the best performance.

Conventional statistical techniques, which are hypothesis driven, have limitations. For instance, only potential risk factors can be selected from data; we cannot use those factors directly to predict NAFLD. Additionally, although there have been prediction models that use conventional statistical techniques, these rely heavily on logistic regression analysis and have limitations. It remains unclear as to which approach has a better calibration and minimizes the errors between the predicted values and the real data. This might lead to a loss of information relevant for outcome prediction. Thus, machine learning techniques were introduced in this study.

Machine learning is a technique for data mining that uses statistical methods to evaluate and analyze data. In our study, classification techniques included 11 ML algorithms. The algorithms were grouped into three families: traditional algorithms, ensemble algorithms, and algorithm extensions. The 6 traditional classification algorithms were kNN, SVM, logistic regression, naïve Bayes, Bayesian networks, and decision tree. AdaBoost, bagging, and random forest (RF) are ensemble learning algorithms. Hidden naïve Bayes (HNB) and aggregating one-dependence estimators are algorithm extensions. The F-measure is regarded as the best evaluation criterion. Precision and recall are both important metrics for FLD prediction since they measure two aspects of quality. If the precision is low, then the user would not use the algorithm due to a high number of false positives. However, if the recall is low, which indicates that the majority of the patients who have FLD were not successfully detected, users would also not use the algorithm. There is a trade-off between precision and recall [16]. One can increase precision by sacrificing recall (and vice versa). One simple way to increase the recall is to predict that all the patients have FLD; in that case the recall would be 1 but the precision would be 0. The F-measure, which is the harmonic mean of precision and recall, is often used to evaluate whether an increase in precision outweighs a loss in recall (and vice versa) [16]. Thus, in our investigation, the F-measure was the most important evaluation metric. For this reason, the Bayesian network achieved the best performance of all the algorithms.

Recent advances in the field of machine learning algorithms have provided us with powerful and promising tools for the study and diagnosis of disease and for the discovery of biomarkers. A prediction model generated by machine learning describes the mapping of a set of attributes to a corresponding class. An important advantage of these algorithms compared with other statistical methods is that machine learning techniques provide a robust multivariate approach with multiple features taken into account simultaneously, without the need for variable selection. Shouval introduced machine learning algorithms for clinical predictive modeling in hematopoietic SCT [42]. Nakayama N established algorithms to predict the prognosis of acute liver failure (ALF) patients through a data-mining analysis to improve the indication criteria for liver transplantation [43].

In our study, the Bayesian network model demonstrated the best performance, which was superior to the widely used logistic regression model. Logistic regression (LR) is a commonly used multivariable method for modeling binary outcomes. However, in real study the linear assumption could not often be satisfied when an ordinary logistic regression was used to explore the real data. Although it can improve the linearity to a certain extent, logarithmic transformation might not correct the linearity when the measures are very large or very small. Therefore, the performance of logistic regression models would certainly be affected by nonlinear circumstances [14, 15]. For these reasons, LR is not always suitable for mathematical analysis. The commonly used FLI and HIS were both formulas derived by logistic regression models [11, 13]. Considering the above explanation, the equations of FLI and HSI could not be suitable. In addition, in our study, FLI reaches a much lower recall and higher precision than ML models, which indicated that FLI can only identify a small number of patients who have NAFLD. Also, FLI and HSI achieve an F-measure lower than Bayesian network model. All of the machine learning models achieve higher F-measure than FLI and HSI.

The Bayesian network model is a graphical model of probabilistic relationships representing the input feature space and label space [44]. It is a directed acyclic graph (DAG), and each node in BN represents a feature or label. A directed edge between two nodes denotes that there is a causal relationship between them. In BN, we denote “parents(a)” as the features or labels on which node “a” would depend. One property of BN is that, given a node “a” and its parents “parents(a)”, “a” is conditionally independent of other nodes not in a ∪ parents(a). The primary goal of a BN-based supervised learning algorithm is to construct the Bayesian network from training data.

In this study, diagnosis of NAFLD was based on ultrasonographic methods, which is not the golden standard to diagnose NAFLD. Ultrasonography is unable to determine the severity of NAFLD. Despite the limitations, ultrasonography is the most commonly used method for population-based studies, with reasonable accuracy. Our results provide important insights into the screening and predictive value of novel artificial intelligence techniques for NAFLD. In future studies, we plan to improve this by including biopsy results to verify the predictive power of ML model.

Given the power of the machine learning approach to process a multiplicity of variables, describe complex nonlinear interactions, and create accurate prediction models, it seems natural to apply it to the complex analysis of the FLD database. Nevertheless, our model has also some limitations, such as the unavailability of liver tissue biopsy data from patients. In future studies, we plan to improve this by including biopsy results. Additionally, there is a lack of model interpretability and standards for data analysis, which are evolving but are areas of ML that require further study.

## 5. Conclusion

Novel machine learning techniques may have screening and predictive value for NAFLD. Applying these novel artificial intelligence techniques may lead to improved experience-based clinical decisions enhancing the early diagnosis rate and reducing end-stage complications.

## Figures and Tables

**Table 1:** Characteristics of study subjects according to presence of NAFLD.

Variables	NAFLD present (n=2522)	NAFLD absent (n=7986)	*Z* value	*P* value
Age (year)	50.86 (12.75)	47.00 (14.96)	11.70^a^	< 0.001
Gender (male/female, n)	1907/615	4971/3015	102.31^b^	< 0.001
Body mass index (kg/m^2^)	26.02 (2.74)	22.49 (2.73)	56.61^a^	< 0.001
Glutamic-pyruvic transaminase (U/L)	23.00(16.00-34.00)	13.00(10.00-19.00)	39.18	< 0.001
Glutamic oxaloacetic transaminase (U/L)	23.00(19.00-30.00)	20.00(16.00-24.00)	25.98	< 0.001
Alkaline phosphatase (U/L)	83.00(71.00-99.00)	77.00(64.25-91.00)	13.88	< 0.001
*γ*-Glutamyltransferase (U/L)	31.00(22.00-47.00)	17.00(13.00-26.00)	39.96	< 0.001
Total bilirubin (*μ*mol/L)	12.90(10.20-16.40)	12.20(9.60-16.10)	5.74	< 0.001
Direct bilirubin (*μ*mol/L)	4.10(3.50-5.10)	3.90(3.20-4.80)	9.7	< 0.001
Indirect bilirubin (*μ*mol/L)	8.80(6.70-11.50)	8.60(6.30-11.30)	3.96	0.092
Total cholesterol (mmol/L)	5.08(4.51-5.72)	4.72(4.17-5.30)	18.06	< 0.001
Triglycerides (mmol/L)	1.63(1.18-2.23)	0.96(0.71-1.36)	40.73	< 0.001
HDL cholesterol (mmol/L)	1.34(1.18-1.53)	1.53(1.32-1.78)	25.86	< 0.001
LDL cholesterol (mmol/L)	2.85(2.35-3.35)	2.60(2.14-3.08)	14.23	< 0.001
Blood urea nitrogen (mmol/l)	4.98(4.24-5.85)	4.93(4.18-5.82)	2.53	0.011
Creatinine (mmol/l)	68.00(59.00-77.00)	66.00(56.00-75.00)	7.51	0.155
Fasting plasma glucose (mmol/L)	5.11(4.75 – 5.65)	4.88(4.57 – 5.24)	18.40	< 0.001
Serum uric acid (*μ*mol/L)	413(63.23)	312.40(53.31)	28.97	< 0.001

Data are expressed as the mean (SD) or median (IQR). ^a^*t* value; ^b^*χ*^2^ value; HDL: high-density lipoprotein; LDL: low-density lipoprotein.

**Table 2:** Risk factors associated with the presence of NAFLD.

**Variables**	**β**	**SE**	**Wald ** **χ** ^2^	***P* value**	**OR (95**%** CI)**
Age (year)	0.018	0.002	59.47	<0.001	1.018 (1.014-1.023)
Body mass index (kg/m^2^)	0.367	0.012	690.14	<0.001	1.443 (1.409 – 1.478)
Glutamic-pyruvic transaminase (U/L)	0.044	0.004	154.629	<0.001	1.045(1.037-1.052)
Glutamic oxaloacetic transaminase (U/L)	-0.032	0.005	38.155	<0.001	0.969(0.959-0.978)
Alkaline phosphatase (U/L)	0.003	0.001	4.013	0.045	1.003(1.000-1.005)
*γ*-Glutamyltransferase (U/L)	0.003	0.001	12.842	<0.001	1.003(1.002-1.005)
Total bilirubin (*μ*mol/L)	0.016	0.005	9.534	0.02	1.016(1.006-1.027)
Triglycerides (mmol/L)	0.400	0.036	125.433	<0.001	1.492(1.391-1.601)
HDL cholesterol (mmol/L)	-0.871	0.117	55.335	<0.001	0.419(0.333-0.527)
Fasting plasma glucose (mmol/L)	0.196	0.028	48.178	<0.001	1.217(1.151-1.286)
Serum uric acid (umol/L)	0.005	0.000	120.929	<0.001	1.005(1.004-1.006)

*β*: partial regression coefficient; SE: standard error of partial regression coefficient; OR: odds ratio; CI: confidence interval; HDL: high-density lipoprotein.

**Table 3:** Discriminative features based on weight scores.

Features	Weight
BMI	0.1980
TG	0.1251
ALT	0.1200
GGT	0.1200
Uric acid	0.0634
AST	0.05156
HDL	0.04769
Glu	0.02777
Age	0.02573
TC	0.02252
LDL	0.01438
ALP	0.0131
Gender	0.01089
DB	0.00878
TB	0.00318
Cr	0.00307
IB	0.00142
Bun	0

**Table 4:** Accuracy, precision, sensitivity, and F-measure values for the 11 algorithms.

**Algorithm **	**Accuracy**	**Specificity**	**Precision**	**Recall (Sensitivity)**	**F-measure**
kNN	80.26%	0.911	0.620	0.459	0.527
SVM	82.73%	**0.946**	**0.725**	0.452	0.557
LR	83.41%	0.934	0.713	0.518	0.600
NB	81.31%	0.913	0.644	0.496	0.560
BN	82.92%	0.878	0.636	0.675	**0.655**
C4.5	80.59%	0.892	0.609	0.534	0.569
AdaBoost	81.01%	0.895	0.620	0.542	0.578
Bagging	82.78%	0.910	0.666	0.567	0.613
RF	82.70%	0.932	0.696	0.496	0.579
HNB	82.42%	0.884	0.630	0.649	0.639
AODE	81.07%	0.852	0.592	**0.680**	0.633
FLI^**∗**^	49.47%	0.812	0.749	0.202	0.318
HIS^#^	54.52%	0.544	0.631	0.448	0.524

^*∗*^The equation of FLI was used to predict NAFLD. A FLI < 30 rules out hepatic steatosis and a FLI ≥ 60 confirms fatty liver [10].

The equation of FLI: FLI= (e^0.953*∗*log⁡(TG)+0.139*∗*BMI+0.718*∗*log⁡(GGT)+0.053*∗*WC−15.745^) /(1 + e^0.953*∗*log⁡(TG)+  0.139*∗*BMI+0.718*∗*log⁡(GGT)+0.053*∗*WC−15.745^)*∗*100.

^#^The equation of FLI was used to predict NAFLD. A HSI of <30.0 rules out NAFLD, while a HSI of >36.0 confirms fatty liver [12].

The equation of HSI: HSI= 8*∗*ALT/AST ratio+ BMI (+2 if DM, +2 if female).

## Data Availability

The data used to support the findings of this study are available from the corresponding author upon request.

## Supplementary Materials

Supplementary Materials This cross-sectional study was performed with data from 10,508 participants who attended an annual health examination in the First Affiliated Hospital, College of Medicine, Zhejiang University, China, in 2010. The Original data was attached in the Supplementary Materials.
